# Association between oxidative stress and postoperative delirium in joint replacement using diacron-reactive oxygen metabolites and biological antioxidant potential tests

**DOI:** 10.1038/s41598-024-80739-x

**Published:** 2024-12-02

**Authors:** Tomoo Yuba, Yoshihisa Koyama, Ayako Takahashi, Yuji Fujino, Shoichi Shimada

**Affiliations:** 1 Department of Anesthesiology and Intensive Care Medicine, Osaka University Graduate School of Medicine, Suita, Japan; 2 Department of Neuroscience and Cell Biology, Osaka University Graduate School of Medicine, 2-2 Yamadaoka, Suita, Osaka 565-0871 Japan; 3 Addiction Research Unit, Osaka Psychiatric Research Center, Osaka Psychiatric Medical Center, Osaka, 541-8567 Japan

**Keywords:** Postoperative delirium, Oxidative stress, Joint replacement surgery, Diacron-reactive oxygen metabolites test, Biological antioxidant potential test, Diseases of the nervous system, Medical research

## Abstract

Postoperative delirium (POD) is a significant complication of surgery that most severely affects older adults and patients with cognitive impairment. This study investigated the relationship between POD and oxidative stress, hypothesizing that increased oxidative stress, measured using diacron-reactive oxygen metabolites (d-ROMs) and biological antioxidant potential (BAP) tests, is associated with the incidence of POD. This prospective cohort study, involving female patients who underwent unilateral or bilateral joint replacement, was conducted at the Osaka University Graduate School of Medicine from June 2022 to July 2023. Blood samples were collected preoperatively and postoperatively to measure oxidative stress markers using the REDOXLIBRA system. The primary endpoint was the association between changes in oxidative stress markers and the occurrence of POD as diagnosed using the Confusion Assessment Method for the Intensive Care Unit and Richmond Agitation–Sedation Scale. Of the 144 patients screened, 60 were eligible, of which 5 developed POD (8.3%). Analysis of oxidative stress markers revealed no significant changes between preoperative and postoperative values of d-ROMs (mean increase + 6.3 ± 54.2 U CARR) and BAP (mean decrease − 37.4 ± 322.9 µM) tests, or BAP/d-ROMs ratio (mean decrease − 0.4 ± 1.7). Further, no significant differences were observed in oxidative stress markers between patients who underwent unilateral and bilateral procedures. However, patients with POD exhibited a significantly higher increase in d-ROMs than those without complications (*p* = 0.015), whereas changes in BAP and BAP/d-ROM ratios were not statistically significant. Although general oxidative stress markers do not significantly change postoperatively, increased d-ROM levels are associated with POD occurrence, indicating that oxidative stress could be a contributing factor to its development. This study underscores the need for further research into specific oxidative markers that may predict POD and guide the development of targeted interventions to prevent this debilitating condition.

**Trial registration** *Name of the registry* Association Between Changes in Blood Oxidative Stress and Postoperative Delirium Following Joint Replacement Surgery: A Retrospective Study. *Trial registration number* 22021. *Date of registration* 6/29/2022. *URL of trial registry record* https://bvits.dmi.med.osaka-u.ac.jp/esct/Apply/project.aspx?PROJECT_ID=6987.

## Introduction

Postoperative delirium (POD), a mental disorder that develops postoperatively, is among the most common complications of anesthesia and surgery^1–3^. Approximately 10% of the surgeries performed under general anesthesia result in POD^4^. Patients who develop POD exhibit higher in-hospital mortality^5–7^, along with increased all-cause mortality during the remote period^6,8^. Notably, old age, preoperative cognitive function, and highly invasive surgery are risk factors for POD development^9,10^. However, little is known about POD pathogenesis, and appropriate prophylaxis and therapy remain lacking. Moreover, a higher number of medical staff is required to manage patients with POD who become excited and violent postoperatively, thereby increasing the burden on the medical facilities. Therefore, elucidating the mechanisms underlying POD and the development of preventive and therapeutic methods are urgently required.

Reportedly, patients who develop POD following cardiac surgery have lower preoperative blood catalase levels and higher postoperative blood superoxide dismutase (SOD) and malondialdehyde levels, suggesting that oxidative stress may be involved in POD pathogenesis^11^. Reactive oxygen species (ROS), byproducts of respiratory metabolism, are essential for immune responses and intracellular signaling and are normally scavenged by in vivo antioxidant enzymes, such as SOD1 and glutathione^12^. In oxidative stress, excess ROS are not removed by the in vivo antioxidant system, which results in cell and tissue damage. Notably, oxidative stress is caused by overeating, prolonged disease, and viral infections. As oxidative stress worsens the onset and progression of many diseases, an accurate understanding of oxidative stress levels is critical. Oxidative stress analyses include measuring oxidized DNA in urine and lipid peroxides in the blood and assessing antioxidant capacity^13^. These analyses are typically performed using blood or urine samples, requiring large sample volumes and special techniques. Recently, diacron-reactive oxygen metabolite (d-ROM) and biological antioxidant potential (BAP) tests have been used to analyze oxidative stress, which can be easily performed using small blood samples. The d-ROMs test estimates the amount of lipid peroxides in the blood, whereas the BAP test estimates the total amount of antioxidants, and the corresponding values are calculated based on the oxidative capacity of hydrogen peroxide and the amount of iron oxide available for reduction, respectively. Oxidative stress refers to an imbalance wherein oxidants exceed the antioxidant capacity^14,15^. The ratio of d-ROMs to BAP (BAP/d-ROMs) is used as a measure of this imbalance and indicates the degree of oxidative stress. These tests are employed to assess metabolic syndrome^16^, smoking^17^, critical illness in intensive care units^18^, lung cancer^19^, and kidney transplant^17^ to clarify the association between oxidative stress and various diseases. These findings may help elucidate the relationship between POD and oxidative stress and develop new therapeutic and preventive methods.

In this study, we performed serum d-ROMs and BAP tests on patients undergoing arthroplasty to investigate whether oxidative stress is involved in the onset of POD.

## Methods

### Study design

Female patients who underwent replacement arthroplasty (knee/hip joint) with blood tests performed the day before and after surgery at Osaka University Graduate School of Medicine between June 2022 and July 2023 were enrolled in this study. Data on height, weight, age, surgical indication, surgical procedure, comorbidities, duration of illness, numeric rating scale score, operation time, and anesthesia method were extracted from the medical records. The primary endpoint was the association between POD and oxidative stress. The secondary endpoints involved were whether postoperative nausea and vomiting (PONV) and the administration of additional analgesics were associated with oxidative stress.

### Ethics statement

This study was approved by the Ethical Review Committee of Osaka University Hospital (Approval No. 22021). Informed consent was obtained from all participants. This study adhered to national ethical guidelines and the Declaration of Helsinki.

### Blood analyses

Preoperative and postoperative blood tests were performed the day before and after the surgery, respectively. Blood tests were usually performed in the clinic, and this study was conducted using surplus specimens (spit samples for biochemical tests). REDOXLIBRA (WISMERLL Co. Ltd., Tokyo, Japan) was used for the d-ROMs and BAP tests.

### POD diagnosis

The Confusion Assessment Method for the Intensive Care Unit (CAM-ICU) and Richmond Agitation–Sedation Scale (RASS) were used to assess delirium once daily postoperatively from the day after surgery (Post operative day, POD1-3). The CAM-ICU is a validated diagnostic algorithm that assesses the key features of delirium by determining changes in mental status, inattention, disorganized thinking, and the level of consciousness^20^. RASS assesses the level of consciousness using a 10-point scale; a negative score indicates decreased reactivity, a score of 0 indicates a calm state, and a positive score indicates excitement^21^. When RASS scores were − 4 or − 5, the CAM-ICU evaluation was suspended, and the assessment was later re-conducted after a period of time. The evaluations in this study were conducted under the responsibility of a specialist in intensive care and anesthesiology.

### Evaluation of PONV and postoperative pain

PONV was defined as the use of antiemetic drugs for nausea from the day after surgery. Postoperative pain was defined as the use of analgesics for wound pain from the postoperative period to the day after surgery.

### Statistical analysis

Data are expressed as the mean ± standard deviation or number (%). Continuous variables were compared using the Student’s t-test. Statistical significance was set at *p < 0.05.

## Results

### Study population and incidence of postoperative complications

Among the 144 patients who underwent arthroplasty at the hospital during the study period, 60 underwent blood tests the day before and after surgery. Patients who underwent preoperative blood tests during outpatient visits before admission and whose blood specimens were discarded and no longer available were excluded. Reports indicate that sex differences in osteoarthritis are marked, with women accounting for approximately 60% of cases, a disparity that becomes even more pronounced after the age of 40^22^. In this study, the average age of participants was around 68 years, and the female proportion of our sample was 78%, aligning closely with these observations. POD occurred in 5 of the 60 patients (8.3%) (Table 1). Patients undergoing arthroplasty are often older and develop POD relatively frequently^23,24^. Further, of the 60 patients, PONV occurred in 27 (45%) patients, and additional analgesics were administered to 38 (63.3%) patients.

**Table 1:** Patient characteristics (*n* = 60).

	*n* = 60
Age (year)	68.7 ± 12.3
Sex	Female 47 (78.3%)
Height (cm)	155.1 ± 9.2
Weight (kg)	55.4 ± 10.6
BMI	23.0 ± 4.0
Primary disease	Knee OA 20 (33.3%), hip OA 17 (28.3%)
Surgical procedure	TKA 19 (31.7%), bi-TKA 1 (1.7%)
	THA 35 (58.3%), bi-THA 5 (8.3%)
Duration of illness (year)	4.0 ± 3.5
Preoperative NRS	6.2 ± 2.2
Preoperative d-ROMs (U CARR)	357.4 ± 86.2
Preoperative BAP (µM)	2130.7 ± 235.9
Preoperative BAP / d-ROMs	6.4 ± 2.0
Anesthesia	GA alone 37 (61.7%)
	GA + epidural anesthesia 4 (6.7%)
	GA + nerve block 19 (31.7%)
Operation time (min)	145.0 ± 67.3
Postoperative delirium	5 (8.3%)
PONV	17 (28.3%)
Pain medication	38 (63.3%)
Postoperative d-ROMs (U CARR)	359.5 ± 83.9
Postoperative BAP (µM)	2093.4 ± 243.8
Postoperative BAP / d-ROMs	6.1 ± 1.2

### Baseline and postoperative oxidative stress markers

**Fig. 1:**
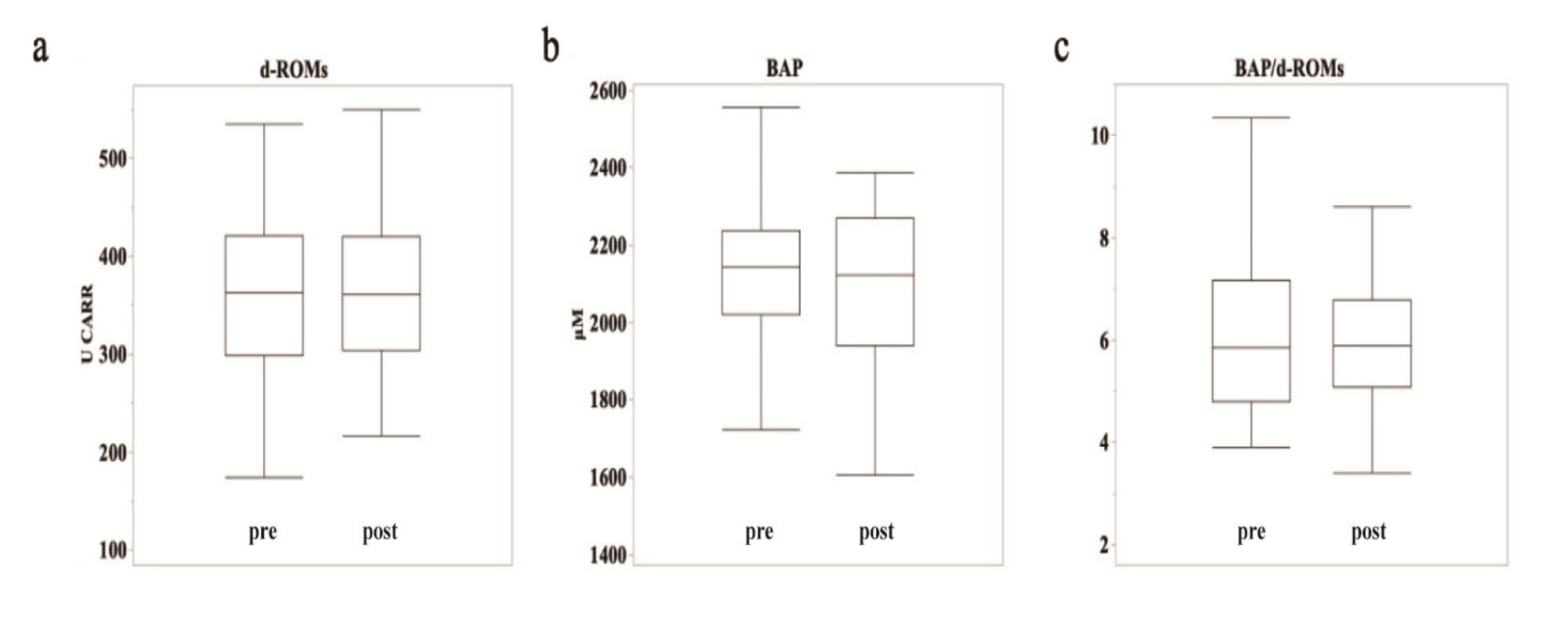
(**a–c**) Box plots of d-ROMs (**a**), BAP (**b**), and BAP/d-ROMs (**c**) before (left) and after surgery (right). The boundary of the box closest to zero indicates the 25th percentile, the line within the box indicates the median, and the boundary of the box farthest from zero indicates the 75th percentile. The whiskers (error bars) above and below the box indicate the 90th and 10th percentiles, respectively.

Preoperatively, d-ROMs and BAP levels were 357.4 ± 86.2 U CARR (mean ± SE) and 2130.7 ± 235.9 µM, respectively, with a BAP/d-ROMs ratio of 6.4 ± 2.0. Postoperatively, d-ROMs and BAP levels were 363.7 ± 81.6 U CARR and 2093.4 ± 243.8 µM, respectively, with a BAP/d-ROMs ratio of 6.0 ± 1.2. The changes in preoperative to postoperative values exhibited an increase of + 6.3 ± 54.2 U CARR for d-ROMs and a decrease of ˗37.4 ± 322.9 µM for BAP, along with a reduction in the BAP/d-ROMs ratio by ˗0.4 ± 1.7 (Fig. 1). Notably, no significant differences were observed in d-ROMs, BAP, or BAP/d-ROMs preoperatively and postoperatively.

### Effect of surgical procedure on oxidative stress

**Fig. 2:**
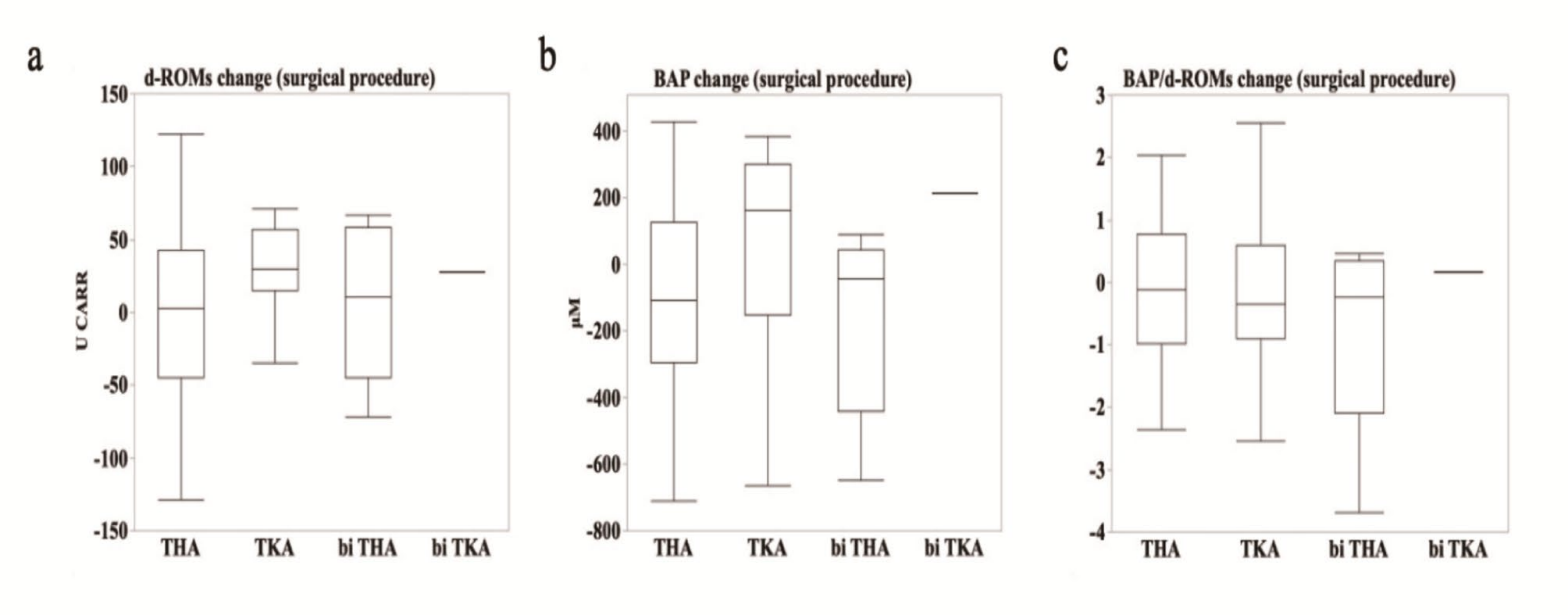
(**a–c**) Box plots of the changes in d-ROMs (**a**), BAP (**b**), and BAP/d-ROMs (**c**) before and after surgery, by surgical procedure. The boundary of the box closest to zero indicates the 25th percentile, the line within the box indicates the median, and the boundary of the box farthest from zero indicates the 75th percentile. Whiskers (error bars) above and below the box indicate the 90th and 10th percentiles, respectively. From left: *THA* total hip arthroplasty, *TKA* total knee arthroplasty, *biTHA* bilateral total hip arthroplasty, *biTKA* bilateral total knee arthroplasty.

We analyzed the variations in oxidative stress markers across different types of joint replacement surgeries to determine whether the type of surgical procedure influences oxidative stress. Subsequently, we compared d-ROMs and BAP values and the BAP/d-ROMs ratio among patients undergoing total hip arthroplasty, total knee arthroplasty, bilateral total hip arthroplasty, and bilateral total knee arthroplasty. Notably, our results revealed no statistically significant differences in the levels of oxidative stress markers between the different surgical groups, despite the varying degrees of surgical invasiveness and tissue trauma associated with each procedure (Fig. 2).

### Impact of postoperative complications on oxidative stress

**Fig. 3:**
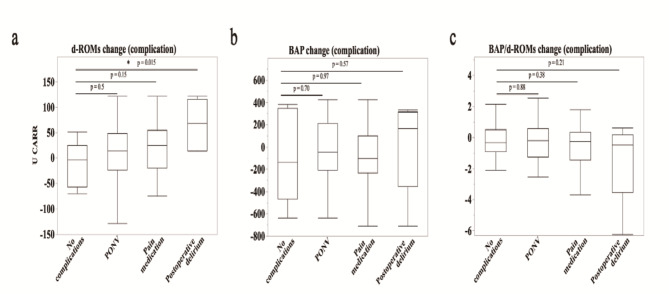
(**a–c**) Box plots of changes in d-ROMs (**a**), BAP (**b**), and BAP/d-ROMs (**c**) before and after surgery, by complication. The boundary of the box closest to zero indicates the 25th percentile, the line within the box indicates the median, and the boundary of the box farthest from zero indicates the 75th percentile. Whiskers (error bars) above and below the box indicate the 90th and 10th percentiles, respectively. From left: no complication; postoperative nausea and vomiting, PONV; pain medication; and postoperative delirium. *p < 0.05 vs. no complications group, determined using a paired Student’s *t*-test.

Finally, we investigated whether the presence or absence of postoperative complications affected oxidative stress. The change in d-ROMs was significantly higher in the POD group (˗10.9 ± 43.2 U CARR) than in the no complications group (65.5 ± 51.0 U CARR, t-test *p* = 0.015) (Fig. 3a). Conversely, no difference was observed in BAP and BAP/d-ROMs ratio between the POD and no complications groups (BAP no complications group: ˗85.4 ± 410.7 µM; POD group: 18 ± 427.2 µM, *p* = 0.57; BAP/d-ROMs no complications group: ˗0.19 ± 1.24; POD group: ˗1.44 ± 2.72, *p* = 0.21) (Fig. 3b, c). Compared with the no complications groups, no significant differences were observed in d-ROMs, BAP, and BAP/d-ROMs in the PONV group (*p* = 0.50, *p* = 0.64, and *p* = 0.88, respectively) or in the group receiving additional analgesia (*p* = 0.15, *p* = 0.88, and *p* = 0.38, respectively).

## Discussion

This study analyzed the relationship between POD and oxidative stress in patients undergoing joint replacement after general anesthesia. POD is an important challenge during recovery following surgery. Notably, many factors, including patient age, preoperative cognitive function, and severity of surgery, are involved in the development of POD^25^; however, the role of oxidative stress in its pathophysiology remains unclear. We explored new biological indicators that indicate the occurrence of POD using d-ROMs and BAP tests, adequately determining oxidative stress.

Contrary to the general trend that oxidative stress increases with more invasive procedures^26^, no significant differences were observed in the oxidative stress markers between unilateral and bilateral joint replacements in our study (Fig. 2). This result suggests that factors other than surgical invasiveness, such as baseline antioxidant capacity and perioperative care, may influence oxidative stress levels. Additionally, this result may be attributed to the limited number of bilateral surgeries analyzed. Therefore, further studies with larger sample sizes are warranted to elucidate the mechanisms underlying POD development. Furthermore, in this study, secondary outcomes such as postoperative nausea and vomiting (PONV) and analgesic use were examined. These factors significantly impact patient recovery, where PONV can increase discomfort and delay the recovery process, and effective pain management is crucial for early recovery and patient satisfaction. While the primary focus of our research was on the relationship between oxidative stress and postoperative delirium, the implications of these secondary outcomes are also pivotal. They directly relate to postoperative management and patient comfort, thus warranting further detailed exploration in future research.

A significant increase in d-ROMs values in patients who developed POD (Fig. 3a) indicated that oxidative stress may be directly involved in POD development. Increased levels of oxidative metabolites in the blood indicate cell and tissue damage, which can lead to neuroinflammation and neurodegeneration^27^. This process can adversely affect brain function, potentially leading to delirium. Surgical procedures impose physical stress on the body, which may induce oxidative stress. Additionally, pre-existing health issues such as cardiovascular disease, diabetes, and dementia can influence the response of the body to postoperative oxidative stress^28^. Collectively, these results suggest that the interplay between oxidative stress and neuroinflammation increases the risk of POD.

In contrast, no significant changes in the BAP values or BAP/d-ROM ratios were observed (Fig. 3b, c). Conversely, the antioxidant capacity of patients has been associated with POD following cardiac surgery^11^. Differences in surgical procedures may contribute to differences in oxidative stress during POD development. Cardiac and joint replacement exhibit distinct oxidative stress profiles and risks of POD. Cardiac surgeries involving cardiopulmonary bypass generate high ROS levels due to prolonged mechanical circulation and oxygenation, potentially increasing the POD risk by increasing systemic oxidative stress^29^. In contrast, although less invasive, joint replacement induces oxidative stress through local tissue damage and inflammation, leading to POD. Additionally, antioxidant enzymes such as superoxide dismutase and catalase may respond differently to each surgical stress, affecting POD development^30,31^. Therefore, understanding the characteristics of the antioxidant capacity responses for each disease is essential for developing POD prevention and management strategies for each surgical procedure. However, we believe that oxidative stress analyses greatly influenced the differences in results. As the BAP test evaluates total antioxidant capacity in vivo, the dynamics of individual antioxidant capacities, such as those of SOD and catalase, are not included. Additionally, antioxidants increase in response to oxidative metabolites; therefore, an increase in antioxidants or depletion due to overutilization may occur if blood is collected later. This is highly conceivable, as the BAP values of the individual samples varied (Fig. 3b). Our clinical study revealed that oxidative stress is involved in the onset of POD. However, whether the increase in oxidative metabolites observed in patients who develop POD is a cause or consequence of the onset of POD is difficult to determine. Nevertheless, this interaction is important for understanding the mechanism underlying POD occurrence, and subsequent research in the future is required. These findings suggest that while elevated d-ROMs may be indicative of increased risk for postoperative delirium, changes in BAP values do not directly correlate with delirium onset. However, it is crucial to consider both markers together in the clinical assessment to fully understand their implications for postoperative delirium risk assessment and management.

In addition, many other physiological and psychosocial factors may be involved in POD development^24,32^. Consequently, in addition to the reduction of oxidative stress, an integrated approach considering these diverse factors is required for the prevention and treatment of POD. Moreover, further research is needed to determine how interventions to reduce oxidative stress affect postoperative outcomes, including the development of POD, in patients. An in-depth understanding of the association between POD and oxidative stress may contribute to the development of effective preventive measures and treatment strategies for POD.

This study had some limitations. First, the diagnostic methods used to evaluate POD may not fully assess all conditions, resulting in a lower reported incidence of delirium than actual. While RASS and CAM-ICU are widely accepted for initial delirium screening, they have limitations in fully assessing delirium, as RASS primarily evaluates consciousness and CAM-ICU is applied for scores from − 3 to + 4. There is a possibility that patients with hypoactive postoperative delirium may have been overlooked in this study. Future research should consider more comprehensive tools to capture all aspects of delirium. It may not be possible to completely eliminate the potential effects of general anesthesia from the previous day in the evaluation on postoperative day one. Additionally, our study included a relatively small sample size, thereby limiting the generalizability of the results. Finally, additional studies with different designs, such as prospective or intervention studies, are required to establish a causal relationship between oxidative stress and POD.

## Conclusion

This study revealed an association between POD and oxidative stress. Our findings provide new perspectives for the prevention and treatment of POD. Notably, POD is a complex multifactorial phenomenon that requires comprehensive management, and future studies will enhance our understanding of POD, facilitating the development of effective countermeasures.

## Data Availability

All relevant data are within this paper.

## Abbreviations

BAP: Biological antioxidant potential
CAM-ICU: Confusion Assessment Method for the Intensive Care Unit
d-ROM: Diacron-reactive oxygen metabolites
POD: Postoperative delirium
PONV: Postoperative nausea and vomiting
RASS: The Richmond Agitation–Sedation Scale
ROS: Reactive oxygen species
SOD: Superoxide dismutase
